# Meta-Prediction of the Effect of Methylenetetrahydrofolate Reductase Polymorphisms and Air Pollution on Alzheimer’s Disease Risk

**DOI:** 10.3390/ijerph14010063

**Published:** 2017-01-11

**Authors:** Suh-Mian Wu, Zhao-Feng Chen, Lufei Young, S. Pamela K. Shiao

**Affiliations:** 1 Department of Nursing, Yuanpei University of Medical Technology, No.306, Yuanpei Street, Hsinchu 30015, Taiwan; suhmian500@gmail.com or suhmian@mail.ypu.edu.tw (S.-M.W.); ivan.chen1966@gmail.com or boivan@mail.ypu.edu.tw (Z.-F.C.); 2 Graduate Institute of Clinical Medical Sciences, Chang Gung University, No.259, Wenhua 1st Rd., Guishan Dist., Taoyuan City 33302, Taiwan; 3 College of Nursing, Augusta University (Previously Georgia Regents University), 987 St. Sebastian Way, EC 4505, Augusta, GA 30912, USA; luyoung@augusta.edu

**Keywords:** meta-analysis, methylenetetrahydrofolate reductase gene, Alzheimer’s disease

## Abstract

*Background*: Alzheimer’s disease (AD) is a significant public health issue. AD has been linked with methylenetetrahydrofolate reductase (*MTHFR*) C677T polymorphism, but the findings have been inconsistent. The purpose of this meta-predictive analysis is to examine the associations between *MTHFR* polymorphisms and epigenetic factors, including air pollution, with AD risk using big data analytics approaches. *Methods and Results*: Forty-three studies (44 groups) were identified by searching various databases. *MTHFR* C677T TT and CT genotypes had significant associations with AD risk in all racial populations (RR = 1.13, *p* = 0.0047; and RR = 1.12, *p* < 0.0001 respectively). Meta-predictive analysis showed significant increases of percentages of *MTHFR* C677T polymorphism with increased air pollution levels in both AD case group and control group (*p* = 0.0021–0.0457); with higher percentages of TT and CT genotypes in the AD case group than that in the control group with increased air pollution levels. *Conclusions*: The impact of *MTHFR* C677T polymorphism on susceptibility to AD was modified by level of air pollution. Future studies are needed to further examine the effects of gene-environment interactions including air pollution on AD risk for world populations.

## 1. Introduction

Alzheimer’s disease (AD) is a degenerative brain disease and the leading cause of dementia [1], thus causing great public health concerns. In 2016, an estimated 5.3 million people in the U.S. were affected by AD, and approximately 10 million U.S. residents will live with AD by 2050 [2]. AD has a devastating impact on not only every aspect of the lives of those affected and their families, but also on society as a whole. In addition, AD is one of the most costly chronic diseases [3]. Between 2015 and 2016, the estimated direct U.S. costs of AD were $236 billion [1], and the indirect costs (e.g., unpaid caregiving, loss or reduction of income, and benefits for caregivers) amounted to another $221.3 billion [4]. To date, the etiology of AD remains unclear, and it is likely a multifactorial disorder involving genetic, environmental, and lifestyle interactions [5,6].

Epidemiology and genome-wide association studies have revealed that the *methylenetetrahydro-folate reductase* (*MTHFR*) gene as one of the candidate genes associated with AD and other neurological conditions (e.g., autism, Down syndrome, seizure, Parkinson’s disease, and stroke) [7,8,9,10]. MTHFR, an enzyme encoded by the *MTHFR* gene, is a key enzyme in reducing the homocysteine level by remethylating homocysteine to methionine. In addition, it plays a vital role in both DNA synthesis and DNA methylation [11]. The *MTHFR* gene, comprised of 11 exons and 10 introns, is located at 1p36.3, on the short arm of chromosome 1 [12]. The two most common polymorphisms in the *MTHFR* gene are C677T (rs1801133) and A1298C (rs1801131). These polymorphisms are associated with *MTHFR* enzyme deficiency, leading to hyperhomocysteinemia. The toxicity induced by an elevated homocysteine level can cause DNA damage and neurotransmitter imbalance, leading to neuronal cell movement dysfunctions and exacerbating neurodegenerative disorders such as AD [13]. Consequently, genome-wide association studies support the impaired MTHFR enzyme activity results in hyperhomocysteinemia, increasing risk of AD and other neurological disorders [9,10,14,15].

Additional evidence indicates that individuals with gene mutations in the methylation pathways are more susceptible to both external (environmental) and internal toxins. New evidence shows that chronic air pollution exposure can contribute to the development of AD and other neurodegenerative disorders through inflammation [16,17] and increased homocysteine levels [18,19]. Moreover, the impaired enzyme activity leads to the accumulation of internal toxins, such as free radicals, which causes the reduction of neurotransmitters by increasing inflammation. As a result, the long-term exposure to air pollution and the presence of gene mutation could act synergistically to increase the prevalence of AD and aggravate the impact of this disease on public health. Compared to least polluted areas, AD death rate increased about 12.1% in highly polluted areas, depending on the types of air pollution (e.g., nitrogen oxides, carbon monoxide, ozone, etc.) [1,20,21,22].

Five previous meta-analyses examined the association between *MTHFR C677T* polymorphism and AD with conflicting results [13,23,24,25]. One study [26] in particular reported the link between *MTHFR* C677T polymorphism and the risk of developing vascular AD. None of these meta-analyses addressed gene-environment interactions. Given that air pollution could be one of the factors involved in AD causality, for its significant impact on public health, the aim of the present study was to conduct a meta-predictive analysis to examine the association between *MTHFR* polymorphisms and AD in the context of gene-environment interaction. Although meta-regression analysis is commonly used to sort through the heterogeneity of the findings in meta-analysis [27], it cannot detect nonlinear patterns and may lead to over-fitted models [28]. To complement the conventional analytics, we utilized big-data analytic techniques (i.e., geographic information system [GIS] maps and recursive partition trees) to visualize distribution and nonlinear patterns of *MTHFR* polymorphisms and AD risk.

## 2. Materials and Methods

### 2.1. Selection Criteria and Identification of Studies

We searched PubMed, PubMed Central, and the Airiti Library to identify all studies that examined the association of *MTHFR* polymorphism and AD risk from year 1998 to June 2016, based on guidelines for preferred reporting meta-analyses of case-control studies (PRISMA) [18,29]. The search was conducted using combinations of the following keywords and subject terms: (“methylenetetrahydrofolate reductase” or “*MTHFR*” or “methylenetetrahydrofolate reductase gene polymorphisms” or “*MTHFR* polymorphisms” or “*MTHFR* variant”) and (“dementia” or “Alzheimer’s disease” or “AD”) and (“case control” or “case-control” or “meta-analysis”). Additionally, we cross checked previous meta-analyses [13,23,24,25,26,30,31,32,33,34,35,36,37] to include all available studies.

We included the articles that: (1) examined the association of the *MTHFR* C677T and A1298C polymorphisms and AD risk using a case-control design; (2) defined AD cases using the criteria developed by the National Institute of Neurological and Communicative Disorders and Stroke (NINCDS) and the Alzheimer’s Disease and Related Disorders Association (ADRDA) [35]; (3) included the genotype frequency in both case and control groups; and (4) were written in English, or non-English ones that provided tables with genotype allele frequencies for both case and control groups. Articles were excluded if they: (1) were not based on case-control design; or (2) did not include complete genotype frequency counts per case and control groups. We searched the previously mentioned databases three times at least 3 months apart until no additional articles were identified.

**Figure 1:**
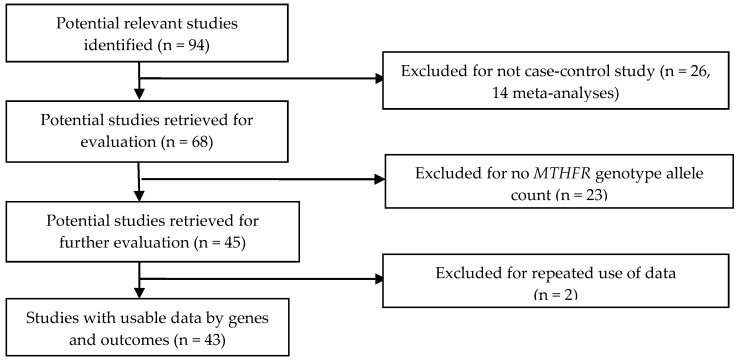
Progression by which studies were selected for this meta-analysis.

Of the 94 identified articles based on the inclusion/exclusion criteria, we excluded 26 that were not case-control studies. We further eliminated 23 articles that lacked genotype allele counts. We then removed two articles for duplicate use of data [38,39]. One publication [40] included two study cohorts (American and Italian). As a result, we included 43 articles with 44 case-control groups in the final analysis (Figure 1, online supplementary Table S1). Study populations were drawn from continents across the globe (Europe, North America, South America, Asia, the Middle East, and Africa). The most investigated racial or ethnic populations in these studies were Asian (24 studies, including four South Asian) followed by Caucasian (13 studies), Middle Eastern (three studies), mixed race (two studies), and African (two studies) (see Table S1). Among these studies, one included participants with vascular Alzheimer’s disease [41]. We present the distributions of *MTHFR* C677T polymorphism per countries for control and AD case groups in Figure S1a, and *MTHFR* A1298C polymorphism for control and AD case groups in Figure S1b.

### 2.2. Data Extraction

Two raters independently extracted the data from the included articles. When there were discrepancies during data extraction, we cross-checked discrepancies and reached consensus among the members.

### 2.3. Quality Assessment

We scored the studies for quality, using criteria appropriate for assessing the quality of meta-analyses [42] and case-control studies [29,43] (Supplementary Table S1). The quality assessment scale included three categories: (1) external validity, with 10 items on demographic data (scores range from 0–11); (2) internal validity, with 12 items on research methods and procedures (scores range from 0–12); and (3) quality reporting (scores range from 0–6). The total scores ranged from 0 to 29, and a higher score showed higher quality [18].

### 2.4. Data Synthesis and Analysis

We used Microsoft Excel (Microsoft Corp, Redmond, WA, USA) to enter data, and StatsDirect Version 3 to perform pooled analyses (2005, StatsDirect, Cheshire, UK). We pooled risk ratios (RR) for the associations of *MTHFR* polymorphisms and AD risk. We used both Cochran’s Q-statistic and I-square (I^2^) to determine the between-study heterogeneity [44], I^2^ statistic is better at assessing inconsistencies across study results regardless of the number of included studies [45]. If the result of the Q test was *p* < 0.05, it indicated the heterogeneity. If there was a significant heterogeneity among the included studies, we used a random effect model [46]. On the other hand, if there was little heterogeneity among the included studies, we used a fixed effect model.

When computing the standardized ratios for RRs, we used the total counts of all three *MTHFR* C677T genotypes (homozygous TT, heterozygous CT, and wild-type CC genotypes), or *MTHFR* A1298C genotypes (homozygous CC, heterozygous AC, and wild-type AA genotypes) as the denominators. Compared to the method using only one of the genotypes as denominator, our approach helped to identify the sources of heterogeneity of the findings [47,48]. Additionally, we examined the sources of heterogeneity using subgroup analyses by geographic regions and other potential contributing factors such as air pollution levels, various AD types, sources of control, and quality score. Further, we used SAS’s JMP 12 program (2013, SAS Institute, Cary, NC, USA) to generate the GIS maps representing geographic patterns and global distributions of *MTHFR* polymorphism and AD risks [49]. Additionally, we used big data analytics including partition trees, nonlinear association curve fit, and heat maps to explore the sources of heterogeneity [18]. The annual death rates from air pollution (AP death) at various geographical areas were reported by the World Health Organization (WHO) as number of deaths per million population (Level 2 = 50–100 deaths/million; Level 3 = 100–250/million; and Level 4 = 250–400 or greater/million) [50].

For the individual studies that reported Hardy-Weinberg Equilibrium (HWE) results, we verified the reported HWE status and reported any discrepancies (Table S1). On studies that showed HWE discrepancies, we performed additional subgroup analyses, and the results confirmed no significant differences between the analyses. Therefore, all studies were included in the final meta-analysis [51]. To detect publication bias, we used Egger’s test and funnel plots [52,53]. An asymmetric plot suggested a possible publication bias and a *p* value of Egger’s test less than 0.05 was considered representative of significant publication bias [54]. To assess the stability of the pooled results, we performed a sensitivity analysis by studies with potential differences such as vascular AD to examine the influence of individual studies on the results.

## 3. Results

### 3.1. Meta-Analysis

For pooled analyses on *MTHFR* C677T polymorphism, we included a total of 4732 AD cases and 5979 controls from 44 study groups (see Table 1). The frequencies of the *MTHFR* homozygous TT genotype were highest in East Asian samples (21.39%), followed by Caucasian (15.76%), Middle Eastern (13.57%), African (11.11%), mixed populations (7.20%), and South Asian (5.08%) (Table 1). For pooled analyses on *MTHFR* A1298C genotypes, we included a total of 564 AD cases and 741 controls from six studies (see Supplementary Table S2). Due to a small sample size, we were unable to identify the different distributions of genotypes on *MTHFR* A1298C across ethnic groups.

Both *MTHFR* C677T TT homozygous and CT heterozygous genotypes were significantly associated with increased AD risk for all samples pooled from 44 study groups (TT genotype: RR = 1.13, 95% CI [1.04, 1.23], *p* = 0.0047; CT genotype: RR = 1.12, 95% CI [1.06, 1.19], *p* < 0.0001). In contrast, *MTHFR* C677T CC wildtype was significantly associated with *reduced* AD risk in all pooled samples (RR = 0.85, 95% CI [0.80, 0.90], *p* < 0.0001). In addition, 677 T allele was associated with *increased* AD risk (RR = 1.12, 95% CI [1.06, 1.17], *p* < 0.0001); and C allele with *reduced* AD risk in all pooled samples (RR = 0.93, 95% CI [0.90, 0.96], *p* < 0.0001). Moreover, the combined CT and TT genotypes were significantly associated with increased AD risk in all pooled samples (RR = 1.11, 95% CI [1.07, 1.16], *p* < 0.0001) (See Table 1). Pooled analyses did not show a significant statistical link between *MTHFR* A1298C mutation and AD risk (see Table S2).

In the subgroup analysis, *MTHFR* C677T TT genotype was associated with increased AD risk in East Asian (RR = 1.19, 95% CI [1.07, 1.32], *p* = 0.0015 for TT genotype), while no significant associations in Caucasian, South Asian, Middle Eastern, African, or mixed populations (Table 1). The *MTHFR* C677T CT heterozygous genotype was associated with *increased* AD risk in South Asian (RR = 1.56, 95% CI [1.25, 1.95], *p <* 0.0001), Middle Eastern (RR = 1.35, 95% CI [1.09, 1.66], *p* = 0.0052), and African samples (RR = 1.50, 95% CI [1.10, 2.04], *p* = 0.0107). On the other hand, the CC wildtype was associated with *reduced* AD risk in Caucasian (RR = 0.91, 95% CI [0.84, 0.99], *p* = 0.0335), East Asian (RR = 0.84, 95% CI [0.78, 0.91], *p <* 0.0001), Middle Eastern (RR = 0.76, 95% CI [0.63, 0.92], *p* = 0.0054), and African samples (RR = 0.66, 95% CI [0.48, 0.92], *p* = 0.0150).

**Figure 2:**
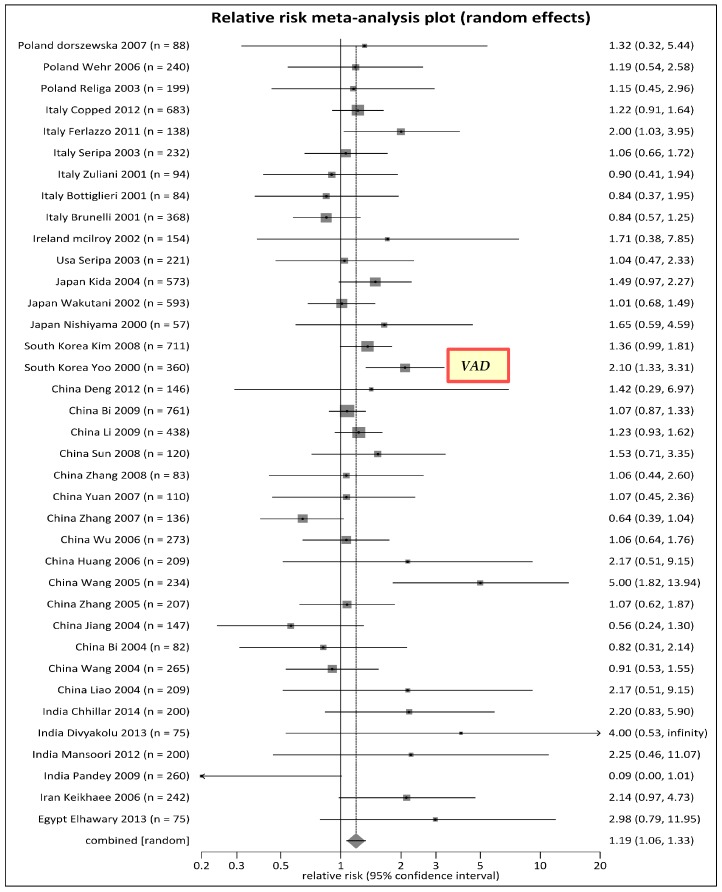
Forest plot for meta-analysis of *MTHFR* C677T polymorphism by TT genotype, countries with risks > 1. Note: VAD: Vascular Alzheimer’s disease in one South Korean Study having RR > 2.

Because of significant heterogeneity across regions, we analyzed subgroups based on: (1) the countries that had *MTHFR* C677T TT genotype as a risk type (RR > 1) (see Figure 2); (2) the countries that had it as a protective genotype (RR < 1) (Supplemental Figure S2a); and (3) others (RR varied around 1) (see Figure S2b). Figure 2 showed that *MTHFR* C677T TT homozygous genotype was a risk type for some European countries (Poland, Italy, and Ireland), U.S., Asian countries (Japan, South Korea, China and India), Iran, and Egypt. While there were heterogeneity in some of these countries including Italy, China, and India, the overall RR for *MTHFR C*677T TT genotype within these countries were greater than 1 when studies within each of these countries were pooled. Therefore, these countries were listed with the countries having RR > 1. Noteworthy, a study from South Korea included vascular AD cases that had higher than average risk of a RR being greater 2, which presented *MTHFR* C677T TT genotype as a potential causal factor of vascular AD. In contrast, *MTHFR* C677T TT genotype was a protective type for other European countries (Sweden and Germany), Israel, and Tunisia (see Figure S2a). Brazil showed mixed results: one study suggested homozygous TT was a protective genotype of AD, while another suggested the harmful effect (see Figure S2b).

### 3.2. Meta-Prediction

**Figure 3:**
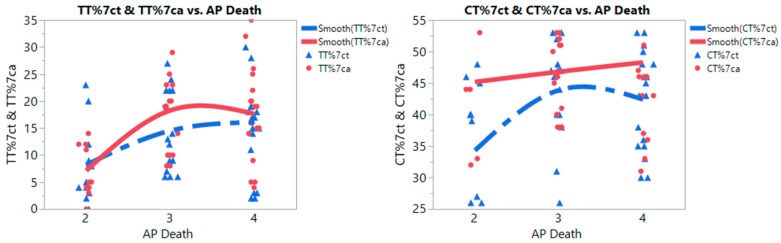
Nonlinear curve fits of *MTHFR* C677T Polymorphism TT (**left**) and CT (**right**) genotypes of control and Alzheimer’s disease (AD) groups with annual death rates from air pollution (AP death) per million population (Levels: 2 = 50–100 deaths/million, 3 = 100–250 deaths, 4 = 250–400+ deaths). Note: TT%7ct: Percentage of *MTHFR* C677T TT genotype in control group; TT%7ca: Percentage of *MTHFR* C677T TT genotype in AD group; CT%7ct: Percentage of *MTHFR* C677T CT genotype in control group; CT%7ca: Percentage of *MTHFR* C677T CT genotype in AD group.

For meta-prediction, we performed both partition tree and Turkey’s test to examine the potential interaction between AP death and *MTHFR* polymorphisms (see Table 2 and Figure 3). We present the partition tree (split groups) and Tukey’s test results side by side for *MTHFR* C677T genotypes and AP death risks in Table 2. The partition tree split the data into two groups by levels of annual AP death rates (Levels, 2 = 50–100, 3 = 100–250, 4 = 250–400 death/million populations). There were significant differences between Levels 2 and 3 (*p* = 0.0021) also Levels 2 and 4 (*p* = 0.0029) for percentage of *MTHFR* C677T TT genotype in the case group. Similarly, there were significant differences between Levels 2 and 3 (*p* = 0.019) also Levels 2 and 4 (*p* = 0.0457) for percentages of *MTHFR* C677T CT genotype in the control group. However, there was no significance for the RRs on the various genotypes (RRCC, RRCT, and RRTT), despite small AICc (smaller is better) on the partition tree analyses. The partition tree and the Tukey’s tests were not performed for MTHFR A1298C genotypes due to small number of studies.

To ensure consistency with other meta-analyses and allow easy comparison, we performed both conventional analyses (Tukey’s test) and big data analytics (partition trees) when examining interaction between gene mutation and air pollution (AP), and its prediction on AD risks. The advanced techniques such as the recursive partition tree, nonlinear fit, and heat maps are able to predict more precisely and accurately by integrating data from diverse sources. The partition-based goodness-of-fit was judged by using the Akaike’s information criterion correction (AICc). A smaller AIC suggests a better model [55]. To compare AICc results with the partition trees, we used the Turkey’s test [56]. All *p* values were two-tailed with a significance level at 0.05. We also used nonlinear fit to examine the associations between AP death, the percentages of each genotype and AD risks. The data distributions were further presented by using a heat map with color spectrum. The nonlinear associations between AP death rates and percentages of *MTHFR* polymorphisms were examined and plotted in Figure 3. With a change in AP deaths from low (Level 2) to high (Level 3 and 4), there was a substantial increase in the percentages of *MTHFR* C677T TT homozygous genotype in both case and control groups (Figure 3, left graph); however higher percentages of *MTHFR* C677T TT genotype were noted in the AD group than that in the control group with increased air pollution levels. On the heat map (Figure S3), a high concentration of *MTHFR* C667T TT genotype appeared in areas with highly polluted air (Level 4 zone) with red blocks. As shown in Figure 3 (right graph), the percentage of *MTHFR* C667T CT heterozygous genotype increased substantially in the control group from low AP death rates (Level 2) to high AP death rates (Levels 3 and 4); however, higher percentages of *MTHFR* C677T CT genotype were noticeable in the AD group than that in the control group with increased air pollution levels.

To detect regional patterns, we used GIS maps generated by the SAS JMP program to visualize the geographic distribution of *MTHFR* C677T polymorphism and AD risk across countries/regions (see Figure S4 for combined TT and CT genotypes, Figure S5 for TT homozygous genotype, and Figure S6 for CT heterozygous genotype). On the third map of each Figures, RRs were presented in chromatic color spectrum with the red color representing AD risk; while the green color standing for protective effects. In Figure S4, combined *MTHFR* C667T TT and CT genotypes were observed as highest risks in Asia, Africa, and South America, followed by North America, then Europe. A similar pattern was observed in Figures S5 and S6. The countries ranking from highest AD risk to protective on *MTHFR* C677T homogenous TT genotype was from Asia including Japan, China, India; then Iran, then Africa including Egypt and Tunisia, then America including Brazil and U.S., and finally Europe including Germany and Sweden. Unlike studies from Germany and Netherlands, studies from Italy, Poland, and Ireland showed significant AD risk in populations with *MTHFR* C677T homogenous TT genotype (Figure S5).

The pooled analyses did not show a statistically significant link between *MTHFR* A1298C polymorphism and AD risk (see Table S2). GIS maps were further generated and demonstrated the potential associations between the geographic pattern of *MTHFR* A1298C polymorphism and AD risk, as well as their impact on AD risk among studies conducted in India, Japan, Tunisia, Poland, Germany, and Brazil (see Figure S7 for combined CC and AC genotypes, Figure S8 for CC homozygous genotype, and Figure S9 for AC heterozygous genotype). The countries with the highest frequency of *MTHFR* A1298C polymorphism (in dark red) in AD cases were India, followed by Tunisia, Poland, Germany, and Brazil (see Figure S7, second map). The countries with highest frequency of *MTHFR* A1298C CC homozygous genotype were India, followed by Japan, Poland, Germany, then Brazil in AD cases (Figure S8, second map). The countries with highest *MTHFR* A1298C heterozygous AC genotype in AD cases were Tunisia, followed by India, Brazil, Germany, and Poland (Figure S9, second map).

## 4. Discussion

Similar to the results reported by previous meta-analyses [13,23,24,25,26] our findings showed a significant association between *MTHFR* C677T polymorphism and AD risk in all samples pooled from 44 study groups with great heterogeneity across geographic areas. To expand the findings from previous meta-analyses, we further conducted meta-prediction to examine potential impact of epigenetic factors, air pollution, on the link between *MTHFR* polymorphisms and AD risks. The nonlinear plots and heat maps demonstrated the percentage of *MTHFR* C677T TT genotype in both case and control groups increased substantially from the regions with low to high levels of air pollution. Compared to control group, higher percentages of *MTHFR* C677T TT and CT genotypes were noticeable for the AD group with the increased level of air pollution. The underlying physiological mechanism of this association pattern could be that global pollution associated with the greenhouse effect may diminish MTHFR enzyme functions and compromise methylation pathways, impairing health in populations with chronic diseases such as AD [18,57]. In addition, studies showed direct effect of air pollution on nervous system. Increasing evidence has implicated air pollution as a chronic source of neuro-inflammation that contributes neuro-degenerative changes in stroke, Alzheimer’s and Parkinson’s diseases [58,59,60].

We further demonstrated the association of *MTHFR* C677T polymorphism with AD risks using big data analytics including GIS maps, and found the risks in countries from Asia (Japan, South Korea, China, and India), as well as Iran and North America (U.S.). Specifically, a study from South Korea included vascular AD cases presented a higher than average RR (2.1) (Table S1), which presented *MTHFR* C677T TT genotype as a potentially significant causal factor of vascular AD based on the criteria commonly used in the international consensus panels. However, mixed results were observed in Europe, Africa and South America. While some studies conducted in Europe (Poland, Italy, and Ireland), African (Egypt) and a mixed population residing in one region of Brazil showed *MTHFR* C677T polymorphism as significant risk factors for AD, other studies conducted in Germany, Sweden, Tunisia, and Brazil had the opposite protective effects. Meta-predictive analyses presented that inconsistent evidence could be explained by variations in the percentages of *MTHFR* C677T polymorphism and the potential gene-environment interactions from air pollution across various geographic areas. The GIS maps further visually demonstrated the source of heterogeneity in the associations between *MTHFR C677T* polymorphism and AD risks, which provided an intuitive insight to the different strengths of linkage between *MTHFR* C677T polymorphism rates and AD risks in regions.

## 5. Conclusions

*MTHFR* C677T polymorphism was associated with increased risk of AD. Epigenetic mechanisms including environmental toxins from air pollution may affect the development of AD through modifying the expressions of genes in the methylation pathways. Additional studies are needed to examine the roles of epigenetic factors in the methylation and metabolism pathways. In the meantime, proactive strategies could be implemented in cities with significant air pollution to prevent AD and promote the health of susceptible populations.

## Figures and Tables

**Table 1:** Pooled meta-analysis: *MTHFR* C677T polymorphism genotypes and risks of AD by race-ethnicity.

Genotype (Number of Studies)	AD Case *N* = 4732 *n* (%)	Control *N* = 5979 *n* (%)	Test of Heterogeneity			Statistical Model	Test of Association	
			Q	*p*	I^2^ (%)		Risk Ratio (95% CI)	*p*
TT (44)	822 (17.37)	925 (15.47)	58.28	0.0600	26.2	Fixed	1.13 (1.04, 1.23)	0.0047
Caucasian (13)	268 (15.76)	219 (14.53)	10.00	0.6158	0	Fixed	1.04 (0.88, 1.22)	0.6605
East Asian (20)	486 (21.39)	633 (18.39)	29.15	0.0636	34.8	Fixed	1.19 (1.07, 1.32)	0.0015
South Asian (4)	15 (5.08)	14 (3.18)	4.24	0.2364	29.3	Fixed	1.36 (0.66, 2.81)	0.4045
Mixed (2)	9 (7.20)	15 (7.11)	2.56	0.1098	60.9	Fixed	1.01 (0.43, 2.33)	0.9906
Middle Eastern (3)	35 (13.57)	33 (13.36)	5.84	0.0539	65.8	Fixed	0.99 (0.64, 1.53)	0.9593
African (2)	9 (11.11)	11 (8.33)	3.34	0.0678	70.0	Fixed	1.14 (0.41, 3.21)	0.8016
CT (44)	2246 (47.46)	2607 (43.60)	67.85	0.0092	36.6	Random	1.12 (1.06, 1.19)	<0.0001
Caucasian (13)	803 (47.21)	659 (43.73)	6.33	0.8986	0	Fixed	1.07 (0.99, 1.15)	0.0864
East Asian (20)	1089 (47.93)	1604 (46.60)	32.15	0.0301	40.9	Random	1.08 (1.00, 1.17)	0.0638
South Asian (4)	118 (40.00)	108 (24.55)	5.74	0.1249	47.7	Fixed	1.56 (1.25, 1.95)	<0.0001
Mixed (2)	71 (56.80)	101 (47.87)	1.46	0.2263	31.7	Fixed	1.21 (0.98, 1.48)	0.0704
Middle Eastern (3)	124 (48.06)	86 (34.82)	1.32	0.5156	0	Fixed	1.35 (1.09, 1.66)	0.0052
African (2)	41 (50.62)	49 (37.12)	0.47	0.4920	0	Fixed	1.50 (1.10, 2.04)	0.0107
CC (44)	1664 (35.16)	2447 (40.93)	61.63	0.0325	30.2	Random	0.85 (0.80, 0.90)	<0.0001
Caucasian (13)	630 (37.04)	629 (41.74)	11.43	0.4926	0	Fixed	0.91 (0.84, 0.99)	0.0335
East Asian (20)	697 (30.68)	1205 (35.01)	21.75	0.2969	12.6	Fixed	0.84 (0.78, 0.91)	<0.0001
South Asian (4)	162 (54.92)	318 (72.27)	19.47	0.0002	84.6	Random	0.78 (0.59, 1.04)	0.0937
Mixed (2)	45 (36.00)	95 (45.02)	0.45	0.5036	0	Fixed	0.78 (0.59, 1.03)	0.0828
Middle Eastern (3)	99 (38.37)	128 (51.82)	1.23	0.5395	0	Fixed	0.76 (0.63, 0.92)	0.0054
African (2)	31 (38.27)	72 (54.55)	0.003	0.9564	0	Fixed	0.66 (0.48, 0.92)	0.0150
T (44)	1945 (41.10)	2228 (37.27)	37.59	0.7044	0	Fixed	1.12 (1.06, 1.17)	<0.0001
C (44)	2787 (58.90)	3751 (62.73)	33.14	0.8608	0	Fixed	0.93 (0.90, 0.96)	<0.0001
CC + CT (44)	3910 (82.63)	5054 (84.53)	70.18	0.0055	38.7	Random	0.98 (0.96, 1.00)	0.0624
TT + CT (44)	3068 (64.84)	3532 (59.07)	70.84	0.0048	39.3	Random	1.11 (1.07, 1.16)	<0.0001
Subgroups								
TT Risk > 1	4062	5205						
TT (37)	761 (18.73)	833 (16.00)	44.31	0.1610	18.8	Fixed	1.19 (1.09, 1.30)	0.0002
CT (37)	1906 (46.92)	2278 (43.77)	57.03	0.0143	36.9	Random	1.10 (1.04, 1.17)	0.0023
CC (37)	1395 (34.34)	2094 (40.23)	56.40	0.0164	36.2	Random	0.84 (0.79, 0.90)	<0.0001
CC + CT (37)	3301 (81.27)	4372 (84.00)	55.24	0.0211	34.8	Random	0.97 (0.95, 0.99)	0.0041
TT + CT (37)	2667 (65.66)	3111 (59.77)	66.60	0.0014	45.9	Random	1.12 (1.07, 1.17)	<0.0001
TT Risk < 1	545	563						
TT (5)	52 (9.54)	77 (13.68)	1.06	0.9000	0	Fixed	0.66 (0.47, 0.92)	0.0142
T (5)	269 (49.36)	228 (40.50)	4.72	0.3177	15.2	Fixed	1.23 (1.07, 1.40)	0.0025
CC (5)	224 (41.10)	258 (45.83)	3.60	0.4630	0	Fixed	0.90 (0.79, 1.04)	0.1552
CC + CT (5)	493 (90.46)	486 (86.32)	0.87	0.9293	0	Fixed	1.06 (1.01, 1.10)	0.0133
TT + CT (5)	321 (58.90)	305 (54.17)	3.60	0.4634	0	Fixed	1.08 (0.97, 1.20)	0.1492
TT Risk varied	125	211						
TT (2)	9 (7.20)	15 (7.11)	2.56	0.1098	60.9	Fixed	1.01 (0.43, 2.33)	0.9906
CT (2)	71 (56.80)	101 (47.87)	1.46	0.2263	31.7	Fixed	1.21 (0.98, 1.48)	0.0704
CC (2)	45 (36.00)	95 (45.02)	0.45	0.5036	0	Fixed	0.79 (0.60, 1.04)	0.0960
CC + CT (2)	116 (92.80)	196 (92.89)	2.76	0.0964	63.8	Fixed	1.00 (0.94, 1.06)	0.9902
TT + CT (2)	80 (64.00)	116 (54.98)	0.13	0.718	0	Fixed	1.18 (0.99, 1.41)	0.0656

*Note.* Data included from 44 studies. Q = Cochran’s Q; AD = Alzheimer’s disease; CI = confidence interval; TT = homozygous mutation; CT = heterozygous mutation; CC = wild type; countries with TT risks > 1: Poland, Italy, Ireland, USA, Japan, South Korea, China, India, Egypt; countries with TT risks < 1: Sweden, Germany, Israel, Tunisia; countries with TT risks varied, ~1: Brazil.

**Table 2:** Meta-prediction: Air Pollution Levels and *MTHFR* C677T Polymorphism Genotypes for Controls (ct) and Alzheimer’s Disease (AD) Cases (ca), and AD Risks.

		Partition Tree				Tukey’s Test					
Variable	AICc	AP Death Levels	Count	Mean	SD	Levels Compared	Difference	SE Difference	Lower CI	Upper CI	*p*
TT%ct	318.732	2	11	8.182	7.427	4/2	7.874	3.319	−0.198	15.945	0.0572
		3 and 4	33	15.364	8.930	3/2	6.3515	3.443	−2.021	14.724	0.1680
						4/3	1.5222	3.032	−5.851	8.896	0.8707
TT%ca	308.987	2	11	7.091	4.549	3/2	11.042	3.089	3.731	18.753	0.0021
		4 and 3	33	17.939	8.441	4/2	10.520	2.978	3.279	17.762	0.0029
						3/4	0.722	2.720	−5.893	7.337	0.9619
CT%ct	317.809	2	11	34.273	10.762	3/2	9.661	3.407	1.377	17.944	0.0190
		4 and 3	33	43.091	7.666	4/2	8.116	3.284	0.130	16.102	0.0457
						3/4	1.544	3.000	−5.751	8.840	0.8646
CT%ca	339.208	2 and 3	26	46.115	11.389	4/2	3.096	4.195	−7.104	13.296	0.7425
		4	18	48.278	9.999	3/2	1.618	4.351	−8.962	12.198	0.9267
						4/3	1.478	3.832	−7.840	10.796	0.9214
CC%ct	366.743	3 and 4	33	41.576	14.331	2/3	16.055	5.959	1.564	30.545	0.0269
		2	11	57.455	16.336	2/4	15.732	5.745	1.762	29.703	0.0242
						4/3	0.322	5.248	−12.440	13.084	0.9979
CC%ca	364.011	4 and 3	33	34.545	12.696	2/4	13.616	5.567	0.078	27.154	0.0484
		2	11	47.727	18.778	2/3	12.661	5.775	−1.382	26.703	0.0845
						3/4	0.956	5.086	−11.412	13.323	0.9807
RRTT	118.544	2	11	1.073	1.016	4/2	0.377	0.342	−0.455	1.209	0.5180
		3 and 4	33	1.424	0.838	3/2	0.321	0.355	−0.542	1.184	0.6413
						4/3	0.057	0.313	−0.703	0.817	0.9821
RRCT	7.777	3 and 4	33	1.124	0.206	2/3	0.250	0.100	0.006	0.493	0.0433
		2	11	1.336	0.359	2/4	0.181	0.097	−0.054	0.415	0.1593
						4/3	0.069	0.088	−0.145	0.283	0.7164
RRCC	−21.842	2 and 4	29	0.822	0.185	3/2	0.026	0.072	−0.149	0.201	0.9299
		3	15	0.848	0.167	3/4	0.026	0.063	−0.128	0.180	0.9132
						4/2	0.0004	0.069	−0.168	0.169	1.0000

*Note. MTHFR =* methylenetetrahydrofolate reductase; CI = confidence interval; AICc = Akaike’s information criterion correction; AP Death Levels = annual death rates from air pollution levels per million population (Levels 2 = 50–100, 3 = 100–250, 4 = 250–400 and greater); RR = Risk Ratio; ct = controls; ca = AD cases; TT%ct = percentages of MTHFRC677T TT genotype in control group; TT%ca = percentages of TT genotype in AD cases; CT%ct = percentages of CT genotype in control group; CT%ca = percentages of CT genotype in AD cases; CC%ct = percentages of CC wildtype in control group; CC%ca = percentages of CC wildtype in AD cases; RRTT = risk ratio of TT; RRCT = risk ratio of CT; RRCC = risk ratio of CC.

## Supplementary Materials

The following are available online at www.mdpi.com/1660-4601/14/1/63/s1. Figure S1. (a) MTHFR C677T percentage of mutations per control and Alzheimer’s (AD) case groups; (b) MTHFR A1298C percentage of mutations per control and Alzheimer’s (AD) case groups. Figure S2. (a) Forest plot for meta-analysis of MTHFR C677T polymorphism by TT genotype, countries with risks <1; (b) Forest plot for meta-analysis of MTHFR C677T polymorphism by TT genotype, countries with risks varied ~1. Figure S3. Heat maps of MTHFR C677T homozygous TT polymorphisms for control and case groups in association with annual deaths from air pollution (TT%7ct: percentage of MTHFR 677 TT in control group; TT%7ca: percentage of MTHFR 677 TT in case group; AP Death: Death rates per million population: Levels 2 = 50–100 deaths, 3 = 100–250 deaths, 4 = 250–400+ deaths). Figure S4. Geographic information maps for percentages of MTHFR C677T TT plus CT genotypes per control and Alzheimer’s disease (AD) case groups, and their associations with AD risks (TTCT%7ct: percentage of MTHFR 677 TT + CT genotypes in control group; TTCT%7ca: percentage of MTHFR 677 TT + CT genotypes in case group; RR7TTCT: the relative risk between percentage of MTHFR 677 TT + CT genotypes and development of AD). Figure S5. Geographic information maps for percentages of MTHFR C677T TT genotype per control and Alzheimer’s disease (AD) case groups, and its association with AD risks (TT%7ct: percentage of MTHFR 677 TT genotype in control group; TT%7ca: percentage of MTHFR 677 TT genotype in case group; RR7TT: the relative risk between percentage of MTHFR 677 TT genotype and development of AD). Figure S6. Geographic information maps for percentages of MTHFR C677T CT genotype per control and Alzheimer’s disease (AD) case groups, and its association with AD risks (CT%7ct: percentage of MTHFR 677 CT genotype in control group; CT%7ca: percentage of MTHFR 677 CT genotype in case group; RR7TT: the relative risk between percentage of MTHFR 677 CT genotype and development of AD). Figure S7. Geographic information maps for percentages of MTHFR A1298C CC + AC genotypes per control and Alzheimer’s disease (AD) case groups, and their associations with AD risks (CCAC%8ct: percentage of MTHFR 1298 CC + AC genotypes in control group; CCAC%8ca: percentage of MTHFR 1298 CC + AC genotypes in case group; RR8CCAC: the relative risk between percentage of MTHFR 1298 CC + AC genotypes and development of AD). Figure S8. Geographic information maps for percentages of MTHFR A1298C CC genotype per control and Alzheimer’s disease (AD) case groups, and their associations with AD risks (CC%8ct: percentage of MTHFR 1298 CC genotype in control group; CC%8ca: percentage of MTHFR 1298 CC genotype in case group; RR8CC: the relative risk between percentage of MTHFR 1298 CC genotype and development of AD). Figure S9. Geographic information maps for percentages of MTHFR A1298C AC genotype per control and Alzheimer’s disease (AD) case groups, and their associations with AD risks (AC%8ct: percentage of MTHFR 1298 AC genotype in control group; AC%8ca: percentage of MTHFR 1298 AC genotype in case group; RR8AC: the relative risk between percentage of MTHFR 1298 AC genotype and development of AD). Table S1. Summary of MTHFR 677 and 1298 loci distributions for included studies on Alzheimer’s disease (AD) by geographic location (43 papers, 44 study groups with genotype counts for control groups). Table S2. Pooled Meta-Analysis: MTHFR A1298C Genotypes and Risks of Alzheimer’s disease (AD).
