# Supplementary material for: Meta-Prediction of the Effect of Methylenetetrahydrofolate Reductase Polymorphisms and Air Pollution on Alzheimer’s Disease Risk
Source: Int J Environ Res Public Health. 2017 Jan 11;14(1):63. doi: 10.3390/ijerph14010063 (PMC5295314; doi:10.3390/ijerph14010063)
Supplement: Supplementary file 1 [file ijerph-14-00063-s001.pdf]

Supplementary Materials: Meta-Prediction of Methylenetetrahydrofolate Reductase Polymorphisms and Air Pollution on Risk of Alzheimer's Disease

Suh-Mian Wu, Zhao-Feng Chen, Lufei Young and S. Pamela K. Shiao

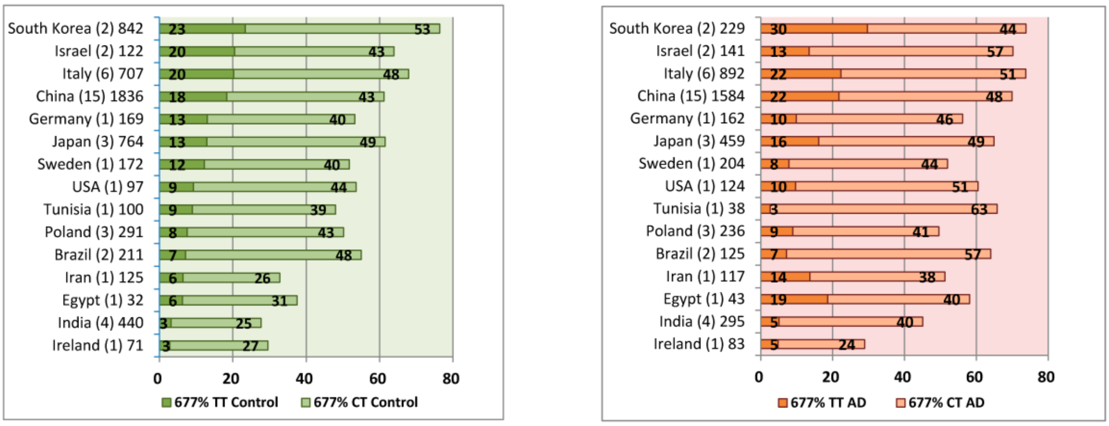

(**a**)

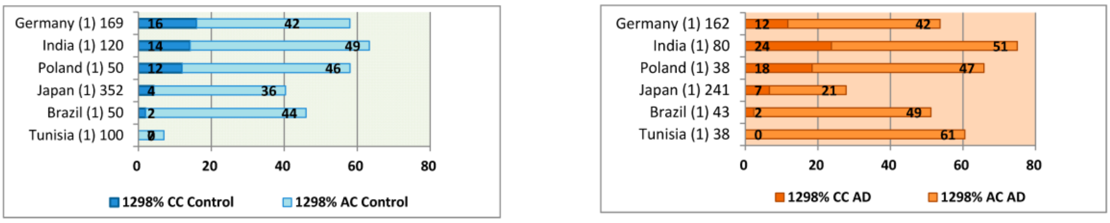

(**b**)

Figure S1. (**a**) *MTHFR* C677T percentage of mutations per control and Alzheimer's (AD) case groups; (**b**) *MTHFR* A1298C percentage of mutations per control and Alzheimer's (AD) case groups.

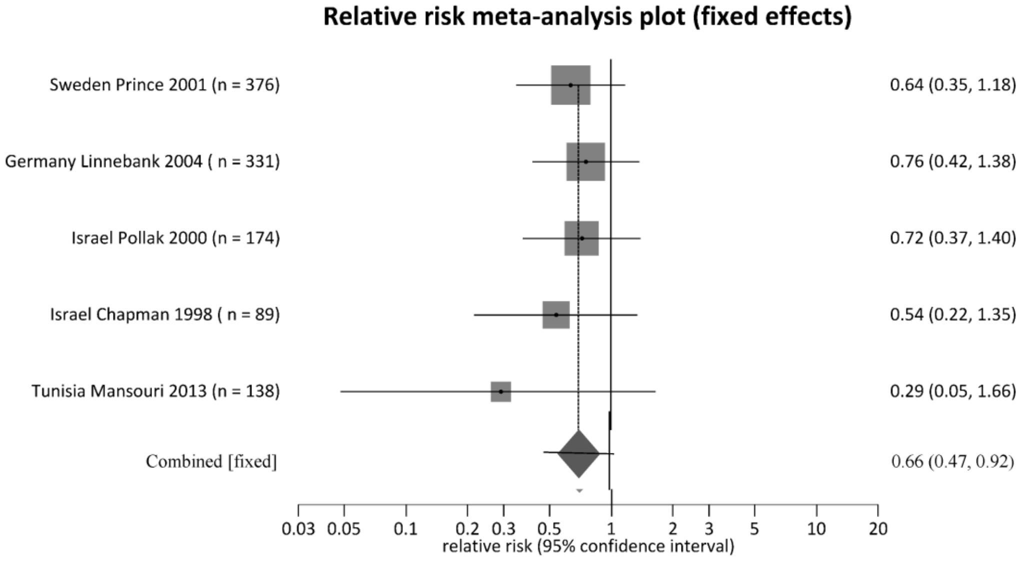

(**a**)

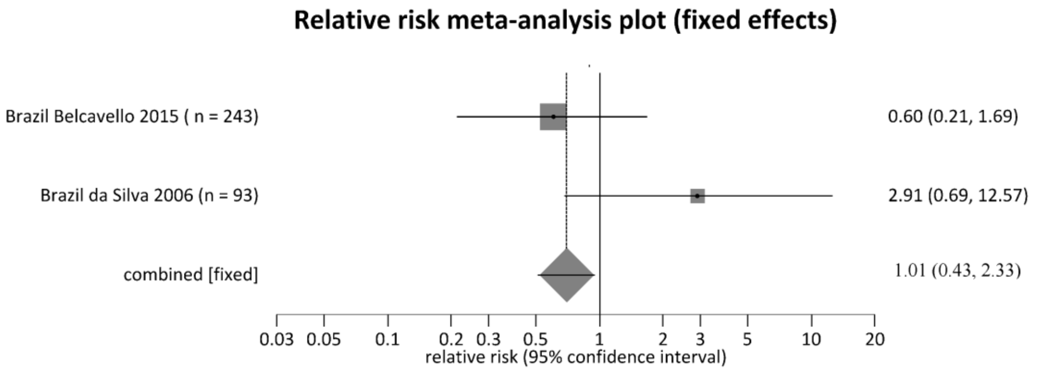

(**b**)

Figure S2. (**a**) Forest plot for meta-analysis of *MTHFR* C677T polymorphism by TT genotype, countries with risks <1; (**b**) Forest plot for meta-analysis of *MTHFR* C677T polymorphism by TT genotype, countries with risks varied ~1.

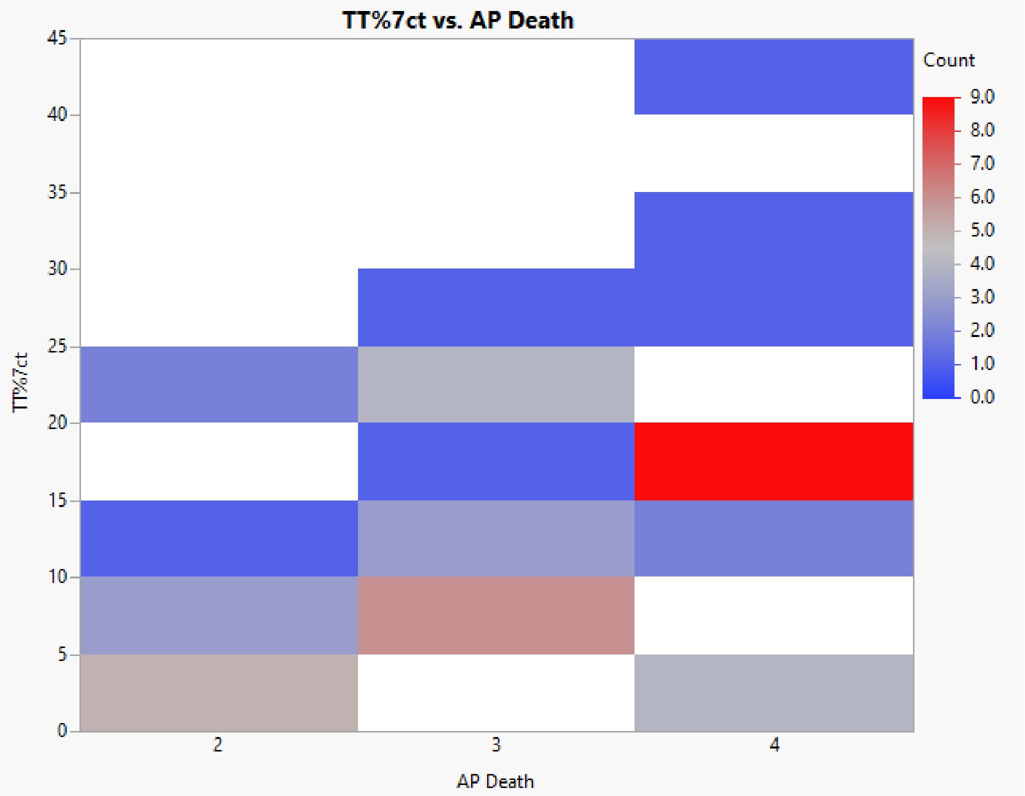

(**a**)

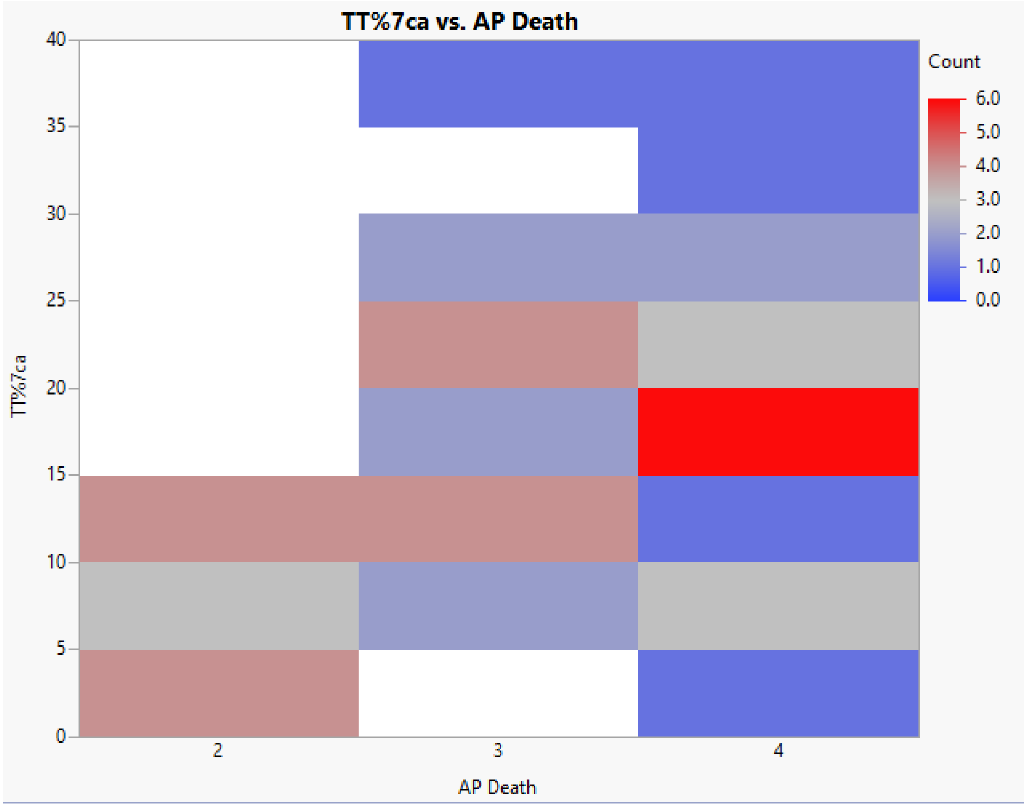

(**b**)

Figure S3. Heat maps of *MTHFR* C677T homozygous TT polymorphisms for control and case groups in association with annual deaths from air pollution (TT%7ct: percentage of MTHFR 677 TT in control group; TT%7ca: percentage of MTHFR 677 TT in case group; AP Death: Death rates per million population: Levels 2 = 50–100 deaths, 3 = 100–250 deaths, 4 = 250–400+ deaths).

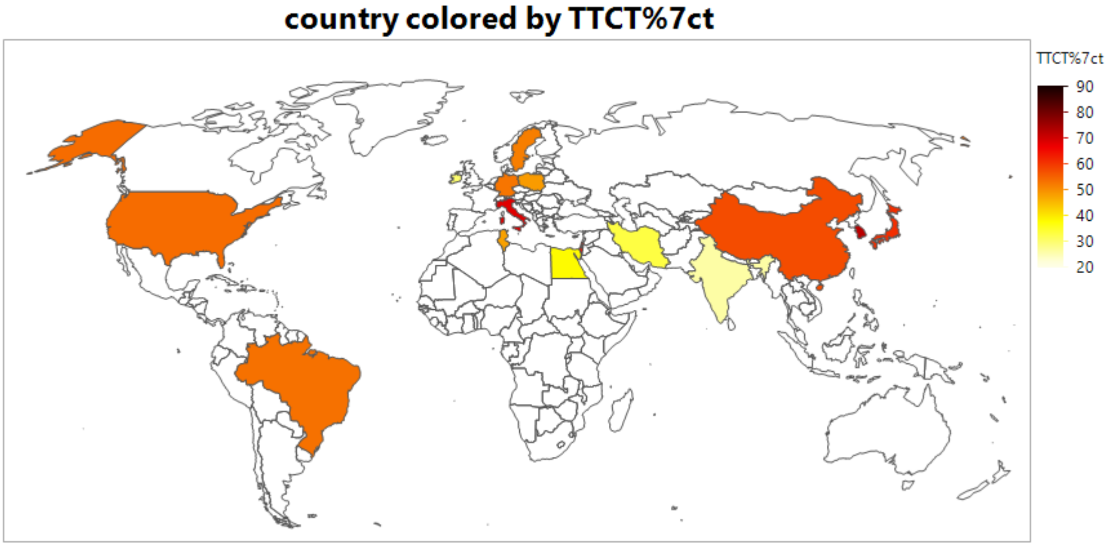

(**a**)

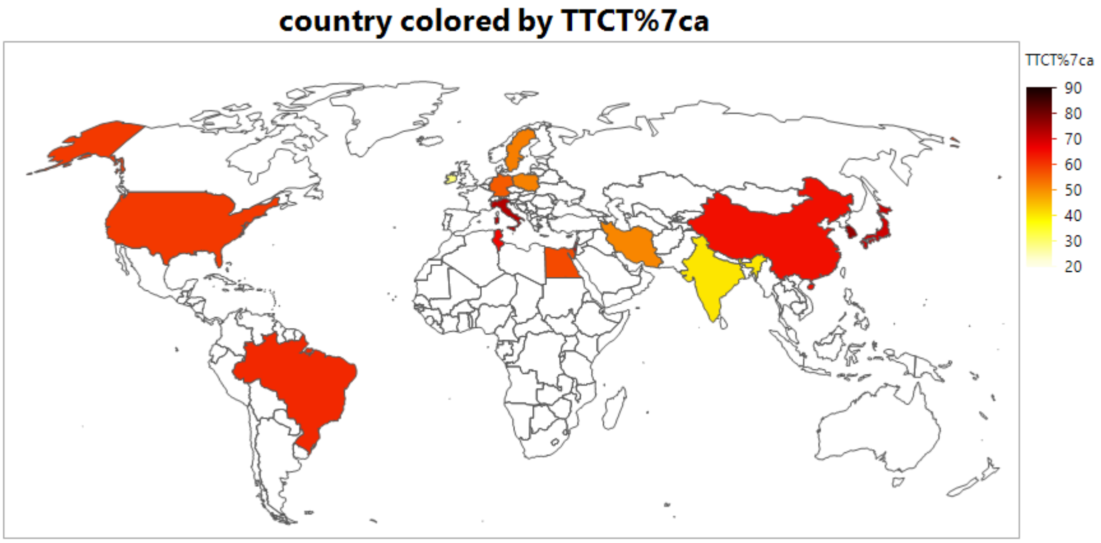

(**b**)

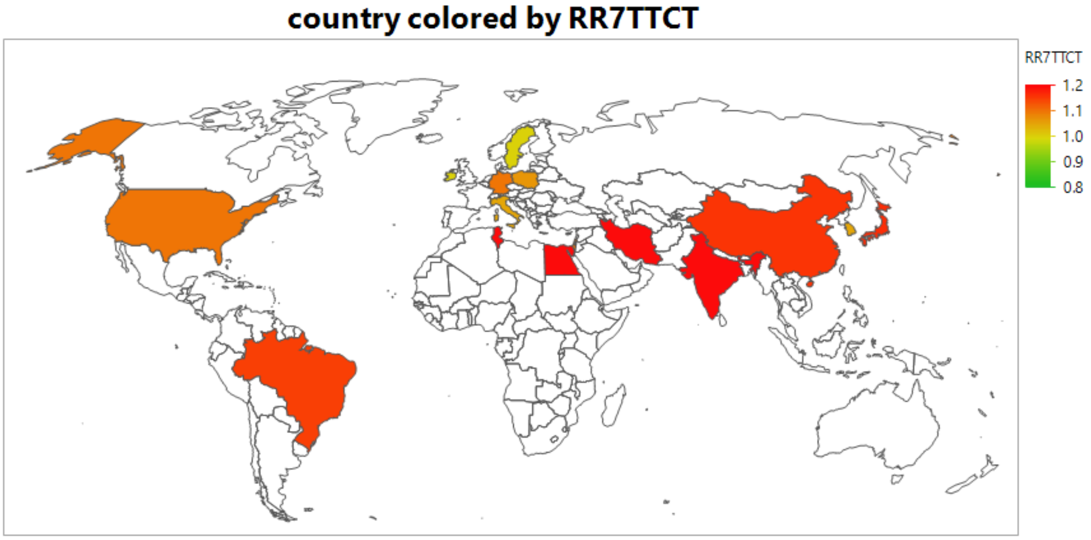

(**c**)

Figure S4. Geographic information maps for percentages of *MTHFR* C677T TT plus CT genotypes per control and Alzheimer's disease (AD) case groups, and their associations with AD risks (TTCT%7ct: percentage of MTHFR 677 TT + CT genotypes in control group; TTCT%7ca: percentage of MTHFR 677 TT + CT genotypes in case group; RR7TTCT: the relative risk between percentage of MTHFR 677 TT + CT genotypes and development of AD).

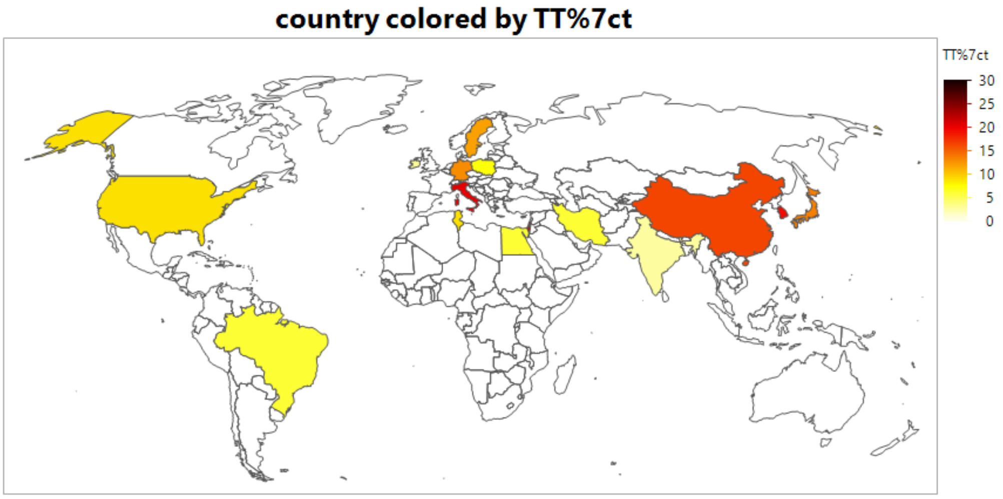

(**a**)

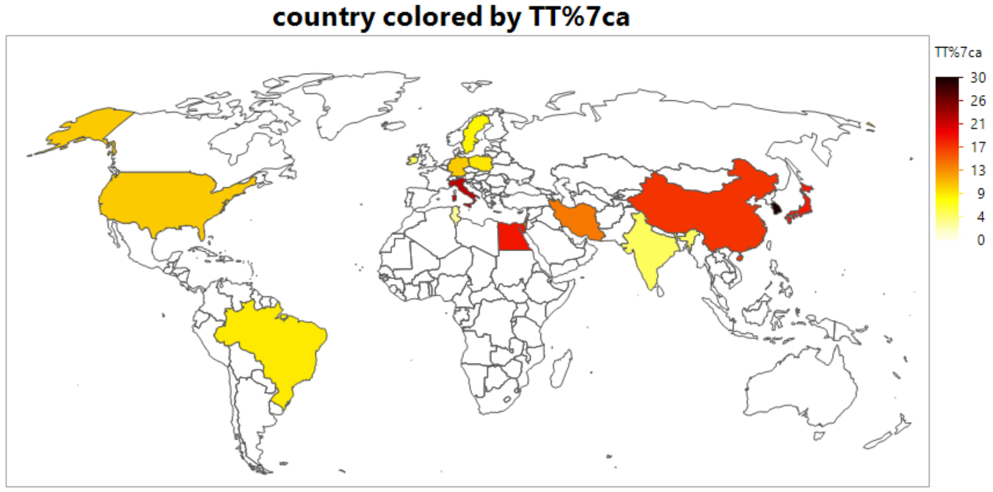

(**b**)

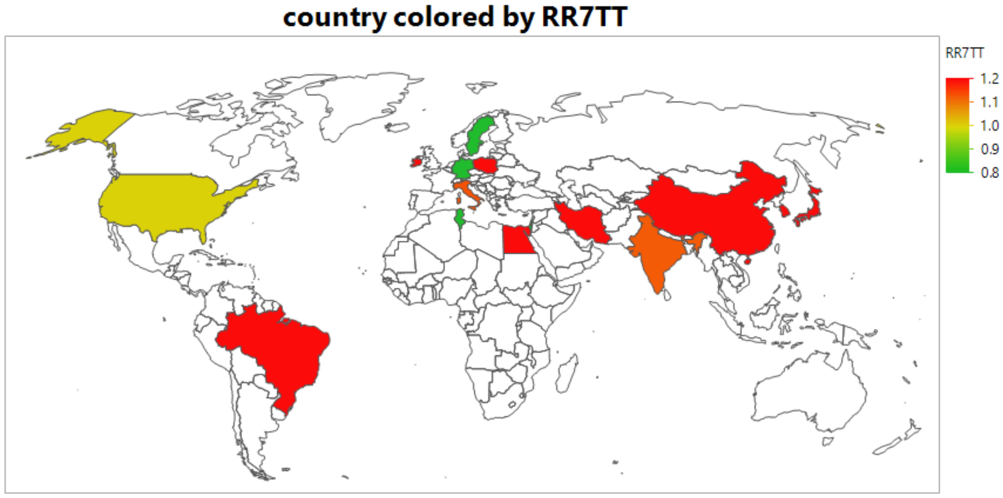

(**c**)

Figure S5. Geographic information maps for percentages of *MTHFR* C677T TT genotype per control and Alzheimer's disease (AD) case groups, and its association with AD risks (TT%7ct: percentage of MTHFR 677 TT genotype in control group; TT%7ca: percentage of MTHFR 677 TT genotype in case group; RR7TT: the relative risk between percentage of MTHFR 677 TT genotype and development of AD).

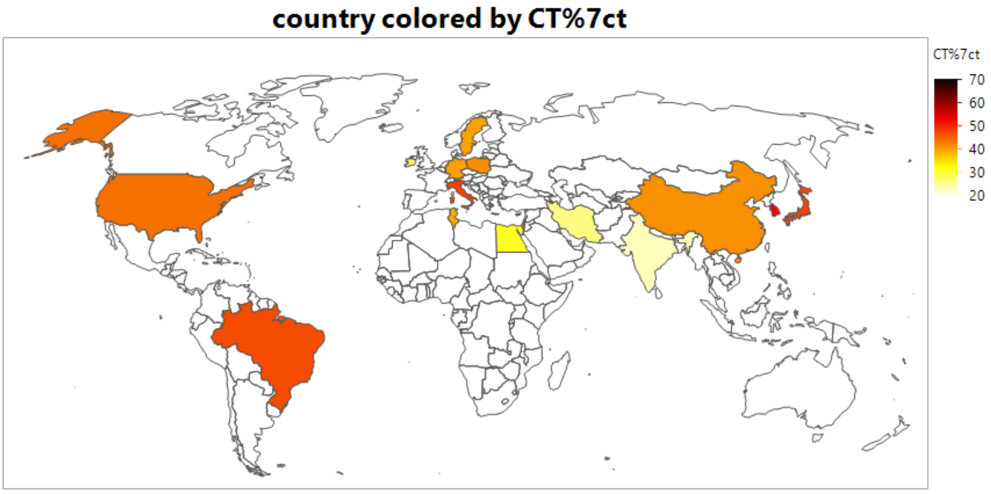

(**a**)

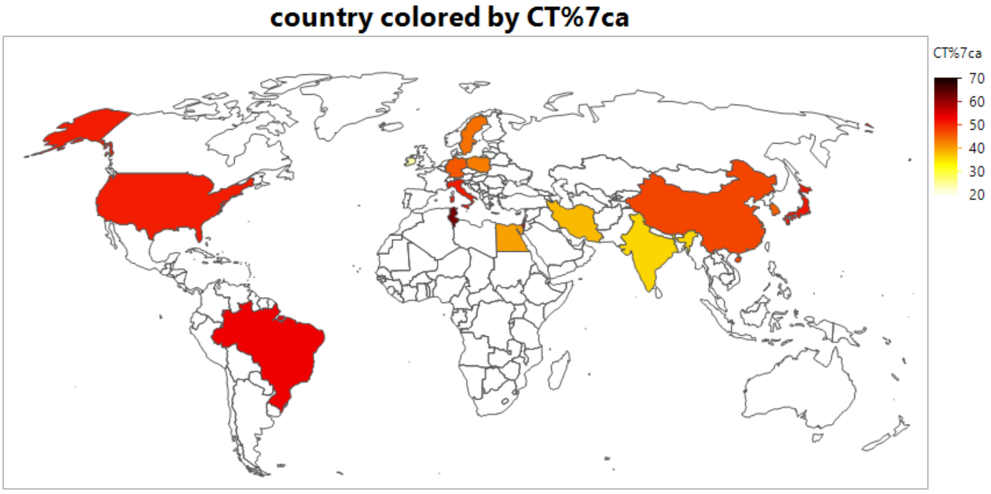

(**b**)

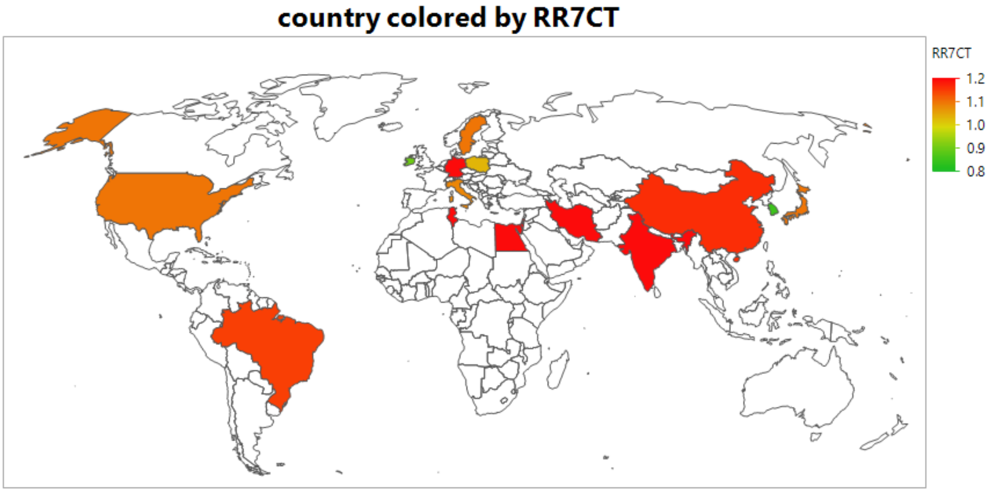

(**c**)

Figure S6. Geographic information maps for percentages of *MTHFR* C677T CT genotype per control and Alzheimer's disease (AD) case groups, and its association with AD risks (CT%7ct: percentage of MTHFR 677 CT genotype in control group; CT%7ca: percentage of MTHFR 677 CT genotype in case group; RR7TT: the relative risk between percentage of MTHFR 677 CT genotype and development of AD).

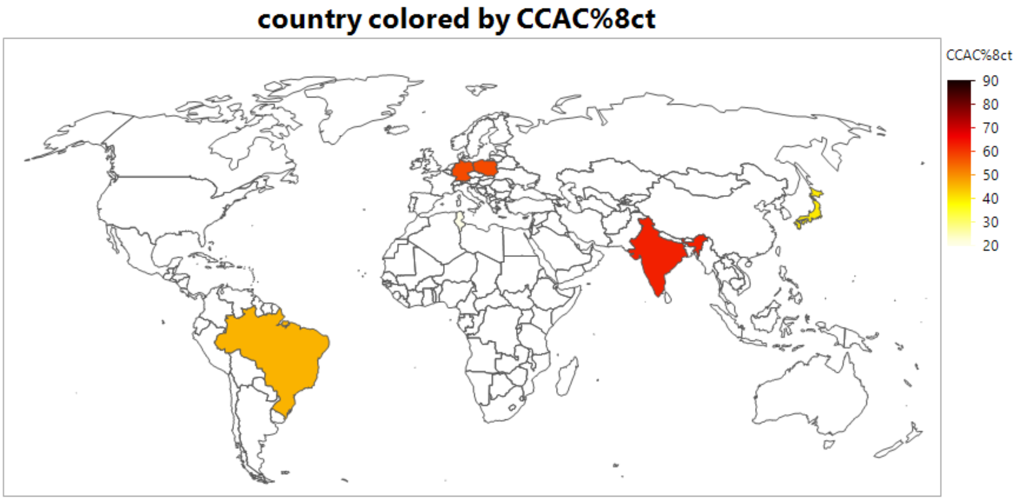

(**a**)

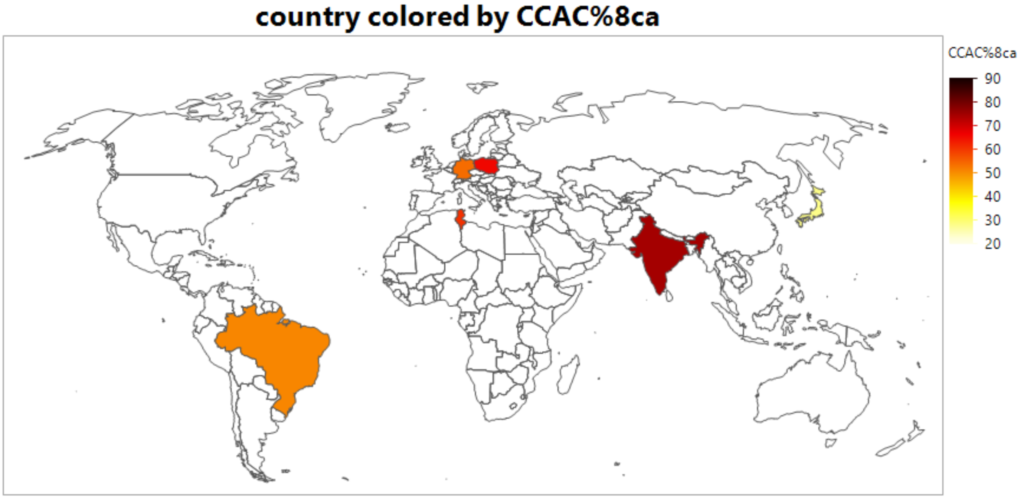

(**b**)

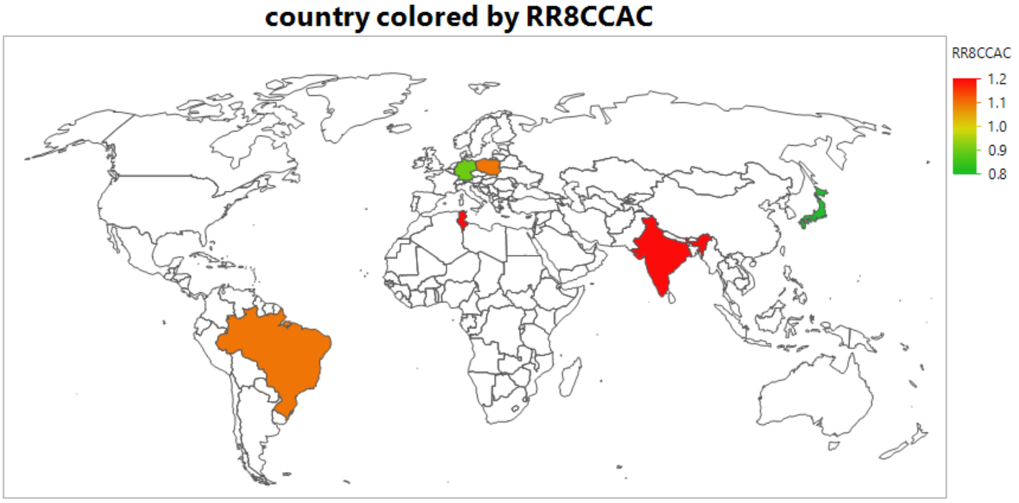

(**c**)

Figure S7. Geographic information maps for percentages of *MTHFR* A1298C CC + AC genotypes per control and Alzheimer's disease (AD) case groups, and their associations with AD risks (CCAC%8ct: percentage of MTHFR 1298 CC + AC genotypes in control group; CCAC%8ca: percentage of MTHFR 1298 CC + AC genotypes in case group; RR8CCAC: the relative risk between percentage of MTHFR 1298 CC + AC genotypes and development of AD).

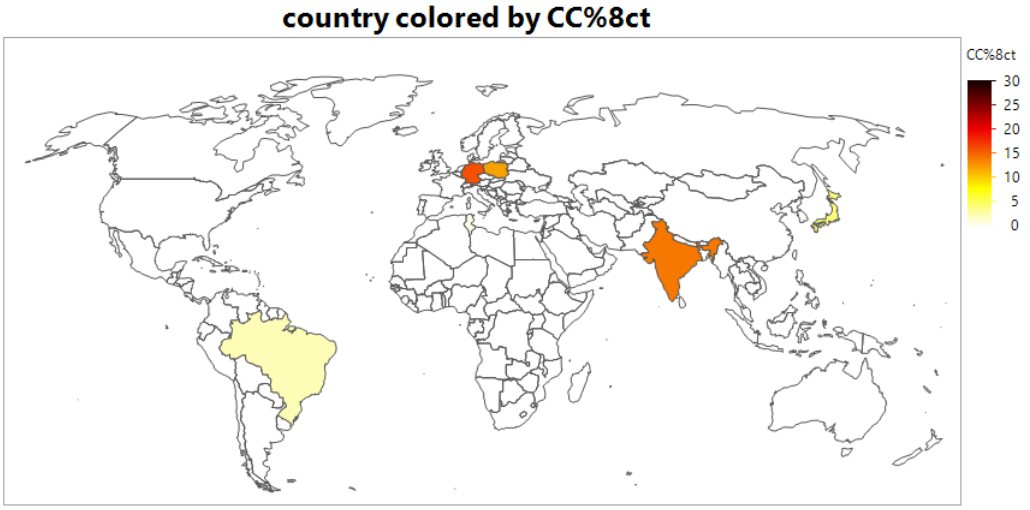

(**a**)

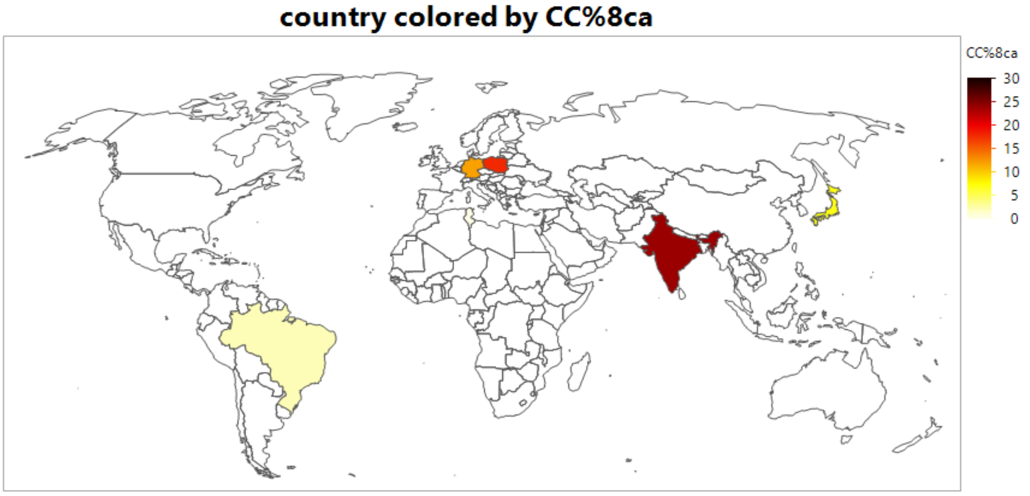

(**b**)

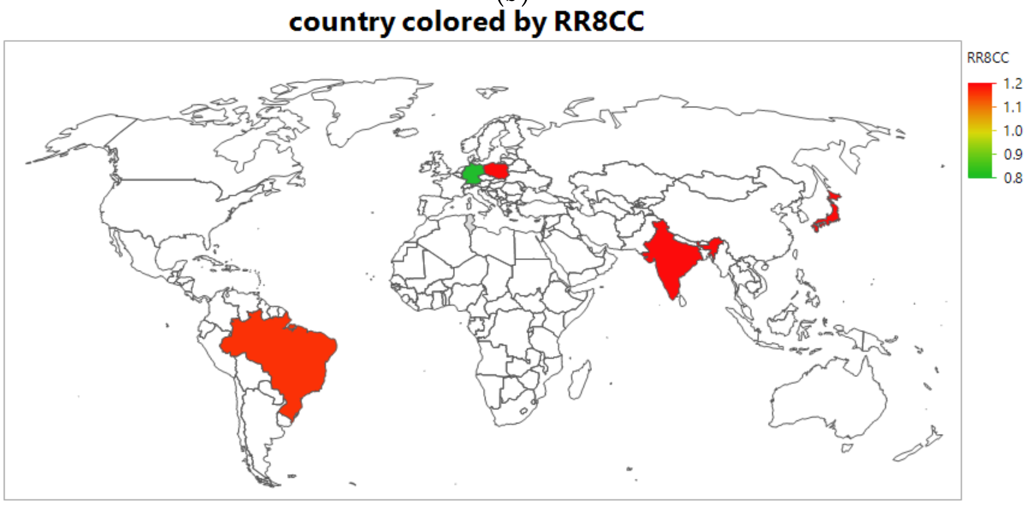

(**c**)

Figure S8. Geographic information maps for percentages of *MTHFR* A1298C CC genotype per control and Alzheimer's disease (AD) case groups, and their associations with AD risks (CC%8ct: percentage of MTHFR 1298 CC genotype in control group; CC%8ca: percentage of MTHFR 1298 CC genotype in case group; RR8CC: the relative risk between percentage of MTHFR 1298 CC genotype and development of AD).

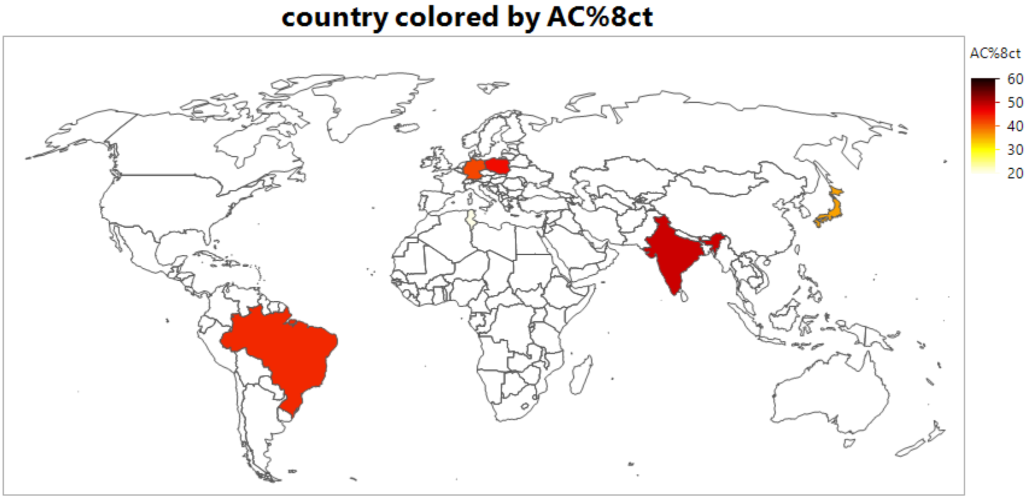

(**a**)

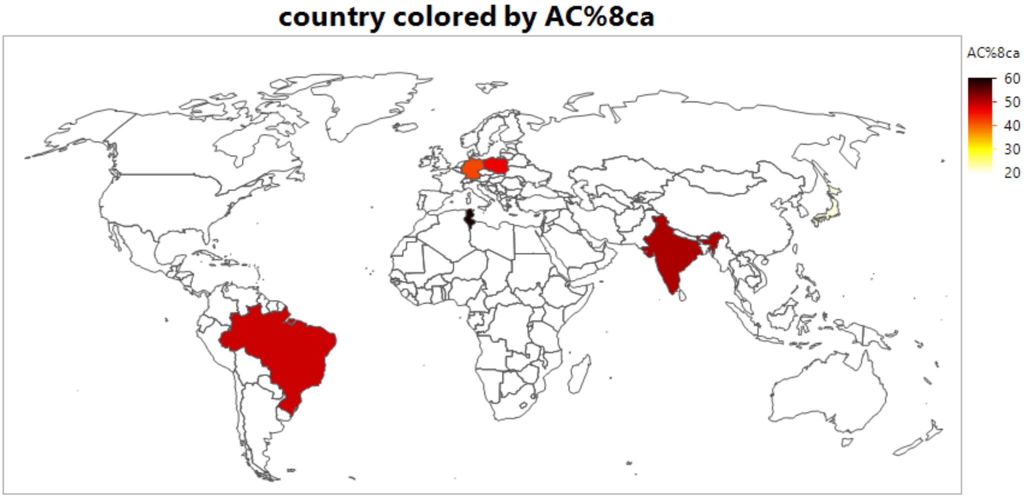

(**b**)

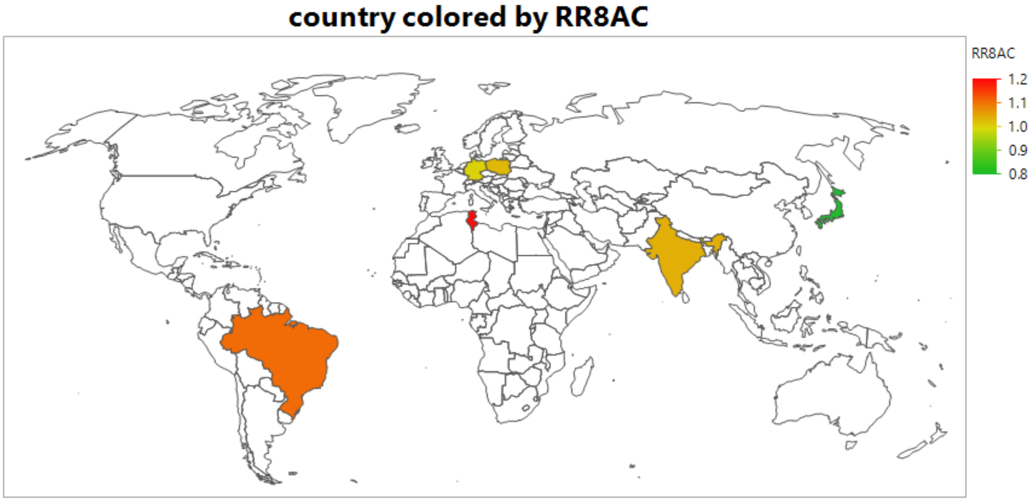

(**c**)

Figure S9. Geographic information maps for percentages of *MTHFR* A1298C AC genotype per control and Alzheimer's disease (AD) case groups, and their associations with AD risks (AC%8ct: percentage of MTHFR 1298 AC genotype in control group; AC%8ca: percentage of MTHFR 1298 AC genotype in case group; RR8AC: the relative risk between percentage of MTHFR 1298 AC genotype and development of AD).

Table S1. Summary of *MTHFR* 677 and 1298 loci distributions for included studies on Alzheimer's disease (AD) by geographic location (43 papers, 44 study groups with genotype counts for control groups).

First Author (Reference Number)	Year	Ethnicity -Country	MTHFR 677									MTHFR 1298							Quality Score
			Cases, Source (sca) *n* (%)				Controls, Source (sct) *n* (%)					Cases, *n* (%)			Controls, *n* (%)				
			SC	CC	CT	TT	SCT	CC	CT	TT	HWE	AA	AC	CC	AA	AC	CC	HWE	
**Europe**																			
Prince [16]	2001	Caucasian -Sweden	1	98 (48.0)	90 (44.1)	16 (7.8)	1	83 (48.3)	68 (39.5)	21 (12.2)	Yes								21 (9, 7, 5)
Dorszewsk [17]	2007	Caucasian -Poland	1	15 (39.5)	20 (52.6)	3 (7.9)	2	27 (54.0)	20 (40.0)	3 (6.0)	Yes	13 (34.2)	18 (47.4)	7 (18.4)	21 (42.0)	23 (46.0)	6 (12.0)	Yes	20 (7, 8, 5)
Wehr [18]	2006	Caucasian -Poland	1	51 (51.5)	38 (38.4)	10 (10.1)	1	63 (44.7)	66 (46.8)	12 (8.5)	Yes								20 (9, 5, 6)
Religa [19]	2003	Caucasian -Poland	1	53 (53.5)	38 (38.4)	8 (8.1)	1	55 (55.0)	38 (38.0)	7 (7.0)	Yes								22 (8, 8, 6)
Linnebank [20]	2004	Caucasian -Germany	1	71 (43.8)	75 (46.3)	16 (9.9)	1	79 (46.7)	68 (40.2)	22 (13.0)	Yes	75 (46.3)	68 (42.0)	19 (11.7)	71 (42.0)	71 (42.0)	27 (16.0)	Yes	15 (9, 2, 4)
Copped [21]	2012	Caucasian -Italy	1	99 (26.2)	193 (51.1)	86 (22.8)	1	106 (34.8)	142 (46.6)	57 (18.7)	Yes								22 (9, 8, 5)
Ferlazzo [22]	2011	Caucasian -Italy	1	17 (24.6)	32 (46.4)	20 (29.0)	2	26 (37.7)	33 (47.8)	10 (14.5)	Yes								21 (8, 8, 5)
Seripa [27]	2003	Caucasian -Italy	1	30 (23.8)	67 (53.2)	29 (23.0)	1	28 (26.4)	55 (51.9)	23 (21.7)	Yes								22 (9, 7, 6)
Zuliani [23]	2001	Caucasian -Italy	1	14 (35.0)	18 (45.0)	8 (20.0)	2	17 (31.5)	25 (46.3)	12 (22.2)	Yes								21 (8, 7, 6)
Bottiglieri [24]	2001	Caucasian -Italy	1	10 (20.8)	29 (60.4)	9 (18.8)	1	11 (30.6)	17 (47.2)	8 (22.2)	Yes								22 (8, 8, 6)
Brunelli [25]	2001	Caucasian -Italy	1	64 (27.7)	120 (51.9)	47 (20.3)	1	39 (28.5)	65 (47.4)	33 (24.1)	Yes								22 (9, 7, 6)
McIlroy [26]	2002	Caucasian -Ireland	1	59 (71.1)	20 (24.1)	4 (4.8)	1	50 (70.4)	19 (26.8)	2 (2.8)	Yes								24 (10, 8, 6)
**North America**																			
Seripa [27]	2003	Caucasian -USA	1	49 (39.5)	63 (50.8)	12 (9.7)	1	45 (46.4)	43 (44.3)	9 (9.3)	Yes								22 (9, 7, 6)
**South America**																			
Belcavello [28]	2015	Mixed -Brazil	1	26 (31.7)	52 (63.4)	4 (4.9)	1	70 (43.5)	78 (48.4)	13 (8.1)	Yes								21 (9, 7, 5)
da Silva [29]	2006	Mixed -Brazil	1	19 (44.2)	19 (44.2)	5 (11.6)	2	25 (50.0)	23 (46.0)	2 (4.0)	Yes	21 (48.8)	21 (48.8)	1 (2.3)	27 (54.0)	22 (44.0)	1 (2.0)	Yes	21 (9, 7, 5)
**Asia**																			
Kida [30]	2004	Asian -Japan	1	64 (33.0)	98 (50.5)	32 (16.5)	1	144 (38.0)	193 (50.9)	42 (11.1)	Yes								22 (8, 8, 6)
Wakutani [31]	2002	Asian -Japan	1	93 (38.6)	112 (46.5)	36 (14.9)	1	137 (38.9)	163 (46.3)	52 (14.8)	Yes	174 (72.2)	51 (21.2)	16 (6.6)	210 (59.7)	127 (36.1)	15 (4.3)	Yes	9 (3, 1, 5)

Nishiyama [32]	2000	Asian -Japan	1	4 (16.7)	14 (58.3)	6 (25.0)	2	13 (39.4)	15 (45.5)	5 (15.2)	Yes								22 (8, 8, 6)
Kim [33]	2008	Asian -South Korea	1	11 (12.8)	43 (50.0)	32 (37.2)	2	122 (19.5)	332 (53.1)	171 (27.4)	Yes								24 (11, 8, 5)
Yoo [34]	2000	Asian -South Korea	2	49 (34.3)	58 (40.6)	36 (25.2)	1	77 (35.5)	114 (52.5)	26 (12.0)	Yes								21 (8, 7, 6)
Deng [35]	2012	Asian -China	1	45 (60.0)	27 (36.0)	3 (4.0)	1	48 (67.6)	21 (29.6)	2 (2.8)	Yes								21 (8, 8, 5)
Bi [36]	2009	Asian -China	1	82 (21.2)	179 (46.4)	125 (32.4)	1	90 (24.0)	172 (45.9)	113 (30.1)	Yes								23 (9, 8, 6)
Li [37]	2009	Asian -China	1	37 (18.7)	92 (46.5)	69 (34.8)	1	69 (28.8)	103 (42.9)	68 (28.3)	No								21 (9, 7, 5)
Sun [38]	2008	Asian -China	1	20 (31.3)	30 (46.9)	14 (21.9)	1	27 (48.2)	21 (37.5)	8 (14.3)	Yes								24 (11, 8, 5)
Zhang [39]	2008	Asian -China	1	21 (48.8)	14 (32.6)	8 (18.6)	1	20 (50.0)	13 (32.5)	7 (17.5)	Yes								21 (8, 8, 5)
Zhang [40]	2007	Asian -China	1	6 (8.8)	44 (64.7)	18 (26.5)	1	11 (16.2)	29 (42.6)	28 (41.2)	Yes								21 (9, 7, 5)
Yuan [41]	2007	Asian -China	1	11 (36.7)	13 (43.3)	6 (20.0)	1	27 (33.8)	38 (47.5)	15 (18.8)	Yes								22 (9, 8, 5)
Wu [42]	2006	Asian -China	1	35 (25.9)	75 (55.6)	25 (18.5)	1	41 (29.7)	73 (52.9)	24 (17.4)	Yes								22 (8, 8, 6)
Huang [43]	2006	Asian -China	1	26 (39.4)	37 (56.1)	3 (4.5)	1	90 (62.9)	50 (35.0)	3 (2.1)	Yes								20 (8, 7, 5)
Wang [44]	2005	Asian -China	1	50 (48.1)	38 (36.5)	16 (15.4)	1	79 (60.8)	47 (36.2)	4 (3.1)	Yes								22 (9, 7, 6)
Zhang [45]	2005	Asian -China	1	39 (37.1)	45 (42.9)	21 (20.0)	2	34 (33.3)	49 (48.0)	19 (18.6)	Yes								21 (9, 7, 5)
Jiang [46]	2004	Asian -China	1	22 (29.3)	46 (61.3)	7 (9.3)	1	24 (33.3)	36 (50.0)	12 (16.7)	Yes								24 (11, 7, 6)
Bi [47]	2004	Asian -China	1	23 (54.8)	13 (31.0)	6 (14.3)	1	21 (52.5)	12 (30.0)	7 (17.5)	No								23 (10, 8, 5)
Wang [48]	2004	Asian -China	1	33 (26.0)	74 (58.3)	20 (15.7)	1	41 (29.7)	73 (52.9)	24 (17.4)	Yes								21 (8, 8, 5)
Liao [49]	2004	Asian -China	1	26 (39.4)	37 (56.1)	3 (4.5)	1	90 (62.9)	50 (35.0)	3 (2.1)	Yes								21 (10, 7, 4)
Chhillar [50]	2014	Asian -India	1	31 (31.0)	58 (58.0)	11 (11.0)	1	69 (69.0)	26 (26.0)	5 (5.0)	Yes								21 (8, 7, 6)
Divyakolu [51]	2014	Asian -India	1	19 (76.0)	5 (20.0)	1 (4.0)	1	42 (84.0)	8 (16.0)	0 (0.0)	Yes								19 (8,5,6)
Mansoori [52]	2012	Asian	1	51	26	3	1	89	29	2	Yes	20	41	19	44	59	17	Yes	21

		-India		(63.8)	(32.5)	(3.8)		(74.2)	(24.2)	(1.7)		(25.0)	(51.3)	(23.8)	(36.7)	(49.2)	(14.2)		(8, 8, 5)
Pandey [53]	2009	Asian -India	1	61 (67.8)	29 (32.2)	0 (0.0)	1	118 (69.4)	45 (26.5)	7 (4.1)	Yes								22 (9, 7, 6)
**Middle-East**																			
Keikhaee [54]	2006	Mid-East -Iran	1	57 (48.7)	44 (37.6)	16 (13.7)	1	84 (67.2)	33 (26.4)	8 (6.4)	Yes								21 (9, 7, 5)
Pollak [55]	2000	Mid-East -Israel	1	30 (32.6)	49 (53.2)	13 (14.1)	1	29 (35.4)	37 (45.1)	16 (19.5)	Yes								21 (8, 7, 6)
Chapman [56]	1998	Mid-East -Israel	1	12 (24.5)	31 (63.3)	6 (12.2)	1	15 (37.5)	16 (40.0)	9 (22.5)	Yes								18 (7, 5, 6)
**Africa**																			
Elhawary [57]	2013	African -Egypt	1	18 (41.9)	17 (39.5)	8 (18.6)	2	20 (62.5)	10 (31.3)	2 (6.3)	Yes								23 (9, 7, 7)
Mansouri [58]	2013	African -Tunisia	1	13 (34.2)	24 (63.2)	1 (2.6)	1	52 (52.0)	39 (39.0)	9 (9.0)	Yes	15 (39.5)	23 (60.5)	0 (0.0)	93 (93.0)	7 (7.0)	0 (0.0)	Yes	21 (8, 8, 5)

Note. Reference numbers refer to the Reference List that follows this table. Sources of cases (SC): 1 = Alzheimer's disease (AD), 2 = Vascular AD; Sources of controls (SCT): 1 = healthy adults, 2 = adults without dementia or AD; HWE = Hardy-Weinberg equilibrium; HWE updated from the original report based on our calculations using the formula available at http://www.koonec.com/k-blog/2010/06/20/hardy-weinberg-equilibrium-calculator; Quality score ranges: Total score 0–29; external validity 0–11; internal validity 0–12; report quality 0–6; NA: Not available.

Table S2. Pooled Meta-Analysis: *MTHFR* A1298C Genotypes and Risks of Alzheimer's disease (AD).

Genotype (Number of Studies)	AD Case (N = 564) n (%)	Control (N = 741) n (%)	Test of Heterogeneity			Statistical Model	Test of Association	
			Q	p	I^2 (%)		Risk Ratio (95% Cl)	p
CC (6)	62 (10.99)	66 (8.91)	5.13	0.2739	22.1	Fixed	1.20 (0.87, 1.66)	0.2550
AC (6)	199 (35.28)	302 (40.76)	44.99	<0.0001	88.9	Random	1.22 (0.79, 1.89)	0.3731
AA (6)	303 (53.72)	373 (50.34)	32.99	<0.0001	84.8	Random	0.84 (0.61, 1.15)	0.2673

Note. Data included from 6 studies. Q = Cochran's Q; CI = confidence interval.
